# Fusion of CTLA-4 with HPV16 E7 and E6 Enhanced the Potency of Therapeutic HPV DNA Vaccine

**DOI:** 10.1371/journal.pone.0108892

**Published:** 2014-09-29

**Authors:** Lili Gan, Rong Jia, Lili Zhou, Jihua Guo, Mingwen Fan

**Affiliations:** 1 Hubei-MOST KLOS & KLOBME, School & Hospital of Stomatology, Wuhan University, Wuhan, Hubei, PR China; 2 Department of Endodontics, School & Hospital of Stomatology, Wuhan University, Wuhan, Hubei, PR China; Georgetown University, United States of America

## Abstract

Preventive anti-HPV vaccines are effective against HPV infection but not against existing HPV-associated diseases, including cervical cancer and other malignant diseases. Therefore, the development of therapeutic vaccines is urgently needed. To improve anti-tumor effects of therapeutic vaccine, we fused cytotoxic T-lymphocyte antigen 4 (CTLA-4) with HPV16 E7 and E6 as a fusion therapeutic DNA vaccine (pCTLA4-E7E6). pCTLA4-E7E6 induced significantly higher anti-E7E6 specific antibodies and relatively stronger specific CTL responses than the nonfusion DNA vaccine pE7E6 in C57BL/6 mice bearing with TC-1 tumors. pCTLA4-E7E6 showed relatively stronger anti-tumor effects than pE7E6 in therapeutic immunization. These results suggest that fusing CTLA-4 with E7E6 is a useful strategy to develop therapeutic HPV DNA vaccines. In addition, fusing the C-terminal of E7 with the N-terminal of E6 impaired the functions of both E7 and E6.

## Introduction

Persistent infection of high-risk human papillomavirus (HR-HPV) causes cervical cancer. Recent studies have considered this condition to be an independent risk factor associated with a subset of anogenital and head and neck cancers. HPV16 is the most prevalent genotype of HPV. It is found in approximately 50% of all cervical cancers. Moreover, HPV16 accounts for more than 90% of HPV-related head and neck squamous cell carcinomas [1], [2].

GARDASIL (quadrivalent, Merck, 2006) and CEVARIX (bivalent, GlaxoSmithKline, 2009) are FDA-approved prophylactic vaccines that contain L1 capsid HPV proteins and that prevent infections with corresponding HPV types [3], [4], [5]. These vaccines can induce high concentrations of neutralizing antibodies, efficiently block the cell entry of high-risk HPVs, and prevent infections. However, they elicit no effect on existing infections and HPV-associated diseases, including cervical cancer. Hence, the development of therapeutic vaccines is urgently needed to control HPV-associated diseases [6].

A commercial therapeutic HPV vaccine remains lacking to date. Numerous therapeutic vaccines against HPV16 or HPV18 have been investigated in preclinical studies or clinical trials over the past decades; these vaccines include peptide/protein, dendritic cell (DC), plasmid DNA, and viral vector-based vaccines [7], [8]. Currently, no therapeutic HPV vaccines have shown significant clinical efficacy against HPV-positive cervical intraepithelial neoplasia (CIN) [9].

E6 and E7 viral proteins induce and maintain malignant transformation by binding to p53 and pRb and disrupting normal cell cycle regulation; hence, these proteins are ideal targets for therapeutic vaccines [10]. Cellular immune responses to HPV are essential to achieve effective treatment. Meanwhile, specific antibodies are also considered to play important roles in tumor rejection [11].

DNA vaccination is an attractive strategy against HPV infection and associated diseases. This strategy is simple, stabile, and capable of inducing both cellular and humoral immune responses. Cytotoxic T-lymphocyte antigen 4 (CTLA-4), expressed on T cell surfaces, is a ligand of B7 molecules on antigen-presenting cells (APCs). We and other groups had demonstrated that fusing CTLA-4 with antigens significantly improves specific immune responses [12], [13], [14], [15]. In the present study, we fused CTLA-4 with HPV16 E7 and E6 as a fusion therapeutic DNA vaccine. This strategy can enhance cellular and humoral anti-HPV16 immune responses and relatively improve anti-tumor effects.

## Materials and Methods

### Ethics statement

This study was approved by the Ethics Committee at the School of Stomatology in Wuhan University.

### Animals and cell lines

Female C57/BL mice (6–8 weeks old) were purchased and maintained in Wuhan University Center for Animal Experiment. HEK-293 cells were grown in Dulbecco's modified Eagle medium (HyClone, USA) supplemented with 10% fetal bovine serum (FBS) and 1% antibiotic-antimycotic (Gibco, USA). TC-1 tumor cells (CCTCC, Wuhan, China) that express HPV16 E6 and E7 proteins were derived from primary C57BL/6 mouse lung epithelial cells. These tumor cells were grown in RPMI1640 (Gibco, USA) supplemented with 10% FBS and 1% antibiotic-antimycotic. DC2.4 cells (kindly provided by Dr. Kenneth Rock in Dana Farber Cancer Institute, Boston, MA, USA) were maintained in RPMI 1640 medium supplemented with 10% heat inactivated FBS and 1% antibiotic-antimycotic.

### Construction of HPV16 E6 and E7 fusion DNA vaccine

**Figure 1:**
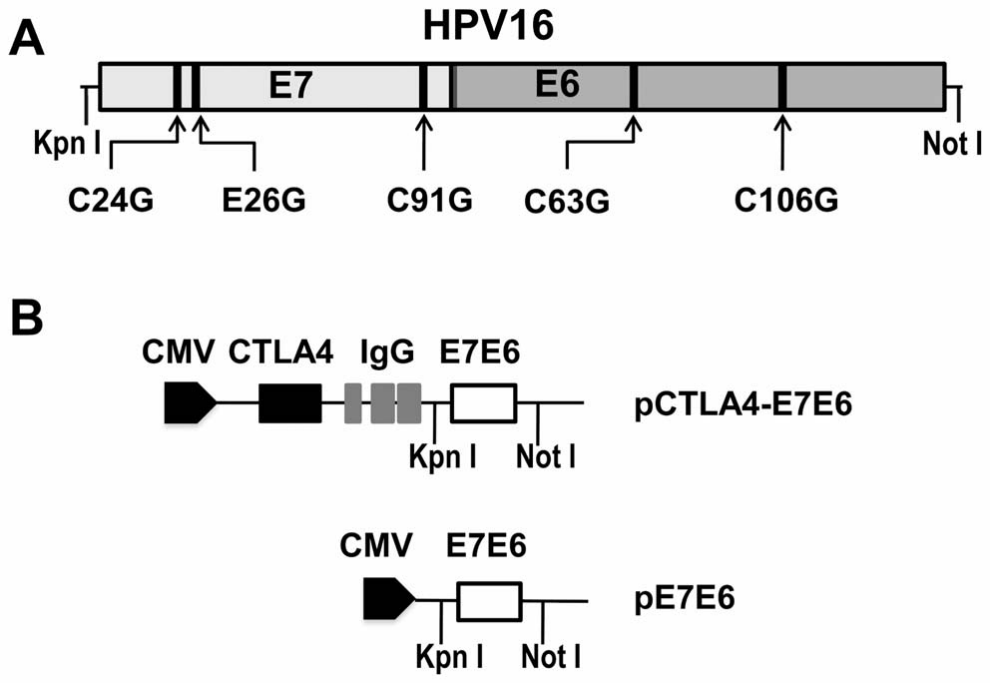
Construction of DNA vaccines pCTLA4-E7E6 and pE7E6. HPV16 E7 gene was fused with E6 gene. The fusion gene was optimized with human biased codons. (**A**) Mutation introduced into HPV16 E7 and E6 to disrupt transformation activity are labeled in the schema. ^24^C, ^26^E and ^91^C of E7 and ^63^C and ^106^C of E6 (arrows) were mutated to glycine. (**B**) pE7E6 was constructed by cloning the modified E7E6 fusion gene into pVAX1 vector at Kpn I and Not I restriction sites. pCTLA4-E7E6 encodes the signal peptide and extracellular domains of CTLA4, the hinge and Fc region of human IgG1, and the E7E6 fusion gene.

To reduce transformation activity, amino acids C_24_ and E_26_ of HPV16 E7 were modified to disrupt the L-X-C-X-E (where X is any amino acid) motif, through which E7 binds to pRb [16], [17], [18]. In addition, C_91_ of the E7 region were modified to reduce transformation activity. In the HPV16 E6 region, amino acids C_63_ and C_106_ were modified to disrupt the C–X–X–C zinc finger binding motif and protect p53 from degradation [19], [20]. All wild type amino acids were mutated to glycine. Codons of the modified E7E6 fusion gene were further optimized with humanized codons to enhance expression and immunogenicity in humans. KpnI and NotI restriction sites were added before the first codon of E7 and after the last codon of E6, respectively. The designed fusion gene fragment was synthesized by Invitrogen Corporation and cloned to pVAX1 vector (Invitrogen, USA) by KpnI and NotI restriction sites. The resulting plasmid was named pE7E6. The pCTLA4-E7E6 vaccine was constructed by cloning the E7E6 fragment into pGJAP/VAX1 [12] at KpnI and NotI sites. The resulting plasmid contains the signal peptide and extracellular domains of CTLA-4, the hinge and Fc region of human IgG1, and the E7E6 fusion gene. Corresponding plasmids carrying wild-type (wt) E7E6 were also constructed and named pwCTLA4-E7E6 and pwE7E6. Two plasmids expressing either wild-type E7 or E6 were constructed and named pwE7 or pwE6, respectively. All plasmids were verified by DNA sequencing (Figure 1). Plasmids were purified by using EndoFree Plasmid Mega Kit (Qiagen, German). The GenBank accession number of the HPV16 reference sequence is NC_001526.2. The exact nucleotide sequence of the fused region between E7 (underlined) and E6 is AGCCAGAAGCCCTTCCAGGACCCC. (Note: the sequence had been optimized with humanized codons.) The protein sizes of CTLA4-E7E6, E7E6, E7 and E6 were predicted to be 83, 39, 17, and 21 kDa, respectively.

### E7E6 expression analysis by Western blot and enzyme-linked immunosorbent assay (ELISA) in cells

**Figure 2:**
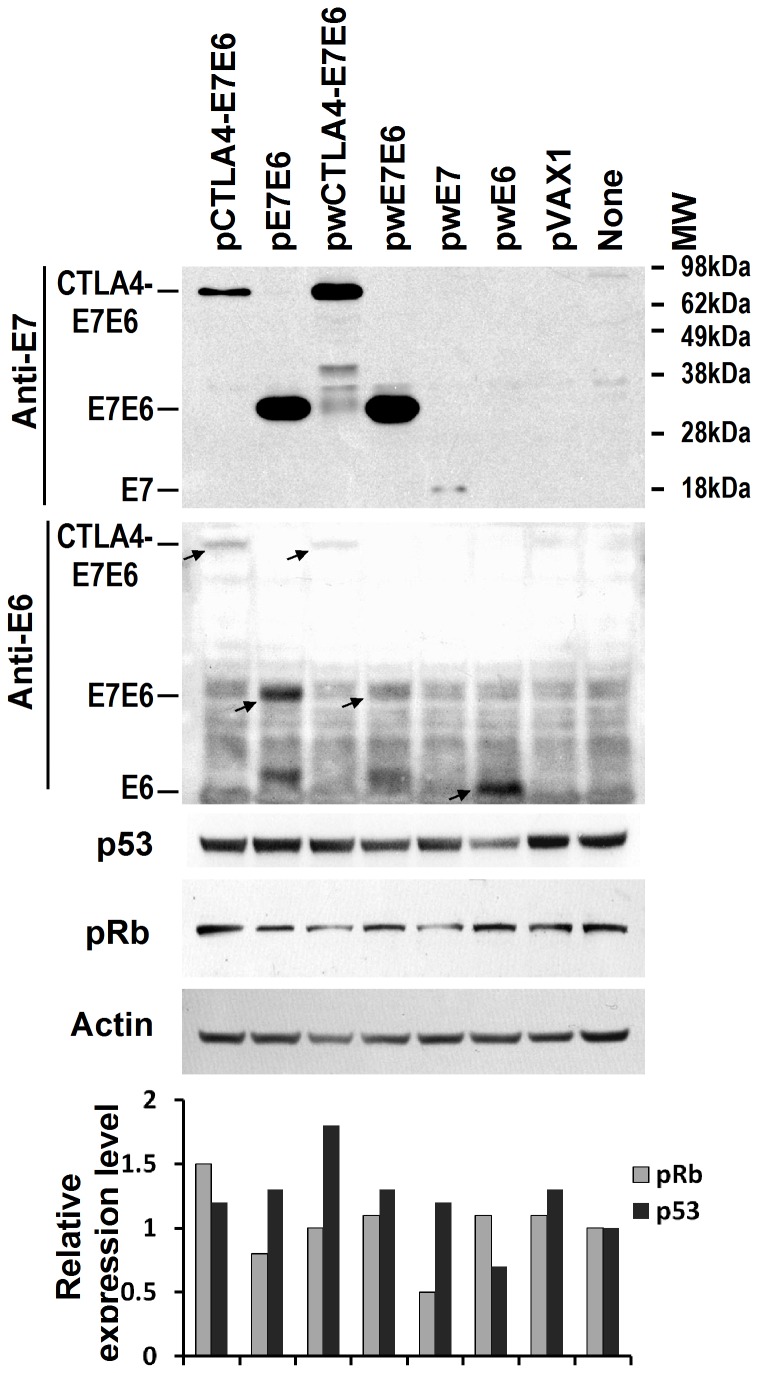
Western blotting analysis of E7E6, p53 and pRb expression. The lysates of 293 cells transfected with pCTLA4-E7E6, pE7E6, pwCTLA4-E7E6, pwE7E6, pwE7, pwE6, pVAX1 or phosphate buffered saline (PBS, labeled as None) were probed with mouse anti-E7, goat anti-E6, mouse anti-p53, rabbit anti-pRb, or mouse anti-β-actin antibody. β-actin served as loading control. pCTLA4-E7E6 encodes CTLA-4 and mutant E7E6 fusion protein. pE7E6 encodes mutant E7E6 fusion protein. Accordingly, pwCTLA4-E7E6 and pwE7E6 carry wild type E7E6 fusion gene. pwE7 and pwE6 encode wild type E7 and E6 respectively. Expressed recombinant proteins were labeled by oblique arrows. MW: molecular weight. The lowest panel showed the relative expression levels of p53 and pRb by normalizing the signal intensities to actin.

A total of 5×10^5^ 293 cells were seeded into six-well plates for 24 hours and then transfected with 2 µg of pCTLA4-E7E6, pE7E6, or pVAX1 in the presence of Lipofectamine 2000 (Invitrogen, USA) according to the manufacturer's instructions. At 48 h after transfection, total protein samples were collected, separated in 10% SDS–PAGE gel, and then blotted with the following antibodies: mouse anti-E7 monoclonal antibody (Santa Cruz, USA), mouse anti-p53 (Ab-6) antibody (Calbiochem, USA), rabbit anti-pRb monoclonal antibody (Epitomics, USA), goat anti-E6 polyclonal antibody (Santa Cruz, USA), or mouse anti-β-actin antibody (Sigma, USA) (Figure 2).

The concentration of recombinant CTLA4-E7E6 fusion protein in the culture medium from the 293 cells transfected with pCTLA4-E7E6 was determined using capture ELISA. Briefly, the medium was serially diluted and added to a microtiter plate precoated with goat anti-human IgG γ chain specific antibody (Sigma) for 2 h. After washing, horseradish peroxidase (HRP)-conjugated goat anti-human IgG antibody (Sigma) was added to the plate. The HRP substrate ortho-phenylenediamine (OPD, Sigma) was used to develop the reaction. Optical density (OD) readings were obtained at 490 nm. The concentration of the CTLA4-E7E6 fusion protein was calculated by interpolation on a standard curve created with standard human IgG antibody (ZSGB-BIO, Beijing, China). Cultured media from 293 cells transfected with pE7E6 or pVAX1 were used as control.

### Flow cytometric analysis (FACS)

FACS was performed to evaluate the binding activity of recombinant CTLA4-E7E6 fusion protein to mouse DC2.4 cells. DC2.4 cells were incubated with the supernatants of cultured media from the 293 cells transfected with pCTLA4-E7E6, pE7E6, or pVAX1. The incubated cells were washed twice with phosphate-buffered saline (PBS) containing 2% FBS (PBS/2%FBS), added with FITC-conjugated goat anti-human IgG (Sigma, USA), and then incubated for 30 min at room temperature. The binding of the CTLA4-E7E6 fusion protein to the cells was analyzed using a BD Biosciences FACSCalibur flow cytometer.

### Therapeutic immunization

Plasmid pCTLA4-E7E6, pE7E6, and pVAX1 for *in vivo* application were vacuum dried (Eppendorf Concentrator 5301) and redissolved in normal saline (NS) to obtain the final DNA concentration at 1 µg/µl.

**Figure 3:**
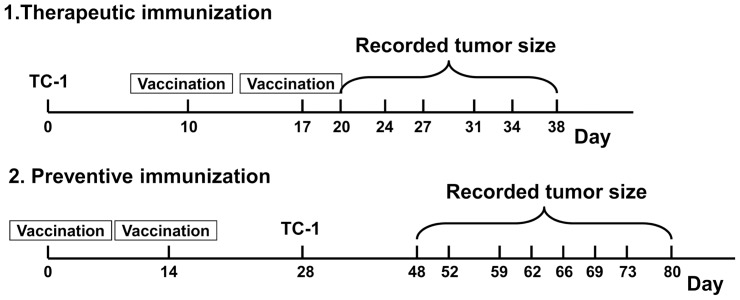
Schematic diagram of vaccination protocols. Therapeutic immunization: mice were challenged with 2×10^5^ TC-1 cells on day 0 and vaccinated twice on day 10 and 17. Preventive immunization: mice were vaccinated on day 0 and day 14, and then challenged with 6×10^4^ TC-1 cells on day 28. Tumor size was recorded twice or thrice a week.

Thirty C57BL/6 mice were randomly divided into four groups. The protocol of therapeutic immunization is shown in Figure 3. TC-1 cells (2×10^5^) in 100 µL of PBS were subcutaneously injected into the left flank of all mice. Group pCTLA4-E7E6 (n = 8) or Group pE7E6 (n = 7) was intramuscularly immunized with 100 µg of pCTLA4-E7E6 or pE7E6 on the left leg on day 10, respectively. Group pVAX1 (n = 8) and Group PBS (n = 7) were respectively immunized with pVAX1 and PBS as controls on day 10. All animals were boosted on day 17. Tumor size was recorded twice or thrice a week. Mice that exhibited tumor ulceration, cachexia, muscle wasting, or inappetence were sacrificed. On day 38, all mice were sacrificed, and spleens were harvested for CTL assay. Serum samples were collected on days 0, 24, and 38. Peripheral blood was collected from the eye vein and then maintained at 4°C overnight. Before collecting blood or sacrificing mice, diethyl ether or chloral hydrate was used to calm and desensitize the mice in both therapeutic and preventive immunization experiments. The serum was obtained by centrifugation at 1000 rpm for 10 min the next day. All samples were stored at −20°C.

### Preventive immunization

A total of 32 C57BL/6 mice were randomly divided into four groups. The protocol of preventive immunization is shown in Figure 3. Group pCTLA4-E7E6 (n = 8) or Group pE7E6 (n = 8) group was intramuscularly immunized with 100 µg of pCTLA4-E7E6 or pE7E6 on the left leg on days 0 and 14, respectively. Group pVAX1 (n = 8) and Group PBS (n = 8) were respectively immunized with pVAX1 and PBS as controls. On day 28, the mice were challenged with 6×10^4^ TC-1 tumor cells in 100 µL of PBS. Tumor size was recorded twice or thrice a week. The serum samples were collected on days 0, 14, 28, and 60. The spleens were immediately harvested after being sacrificed on day 80.

### CTL assay

Spleen cells were harvested from vaccinated mice, and single cell suspensions were prepared using 40 µm cell strainer (BD Falcon, USA). Red blood cells were lysed with RBC lysis buffer (Sigma, USA) for 6 min and then washed twice with HBSS (HyClone, USA). Spleen cells were resuspended to 1×10^7^ cells/mL with RPMI 1640 (HyClone, USA) containing 10% inactivated FBS.

The CTL assay was performed by using the CytoTox 96 nonradioactive cytotoxicity assay (Promega, USA) based on the detection of lactate dehydrogenase (LDH) release. Briefly, spleen cells were cultured in RPMI 1640 containing 50 units/mL IL-2 (Peprotech, USA) at 37°C for 12 h. The next day, 1×10^7^ cells were harvested and co-cultured for 5 d with 2×10^6^ TC-1 cells pretreated with 50 µg/mL mitomycin C (Sigma, USA) for 1 h at 37°C in six-well plates. The spleen cells were collected by centrifugation and then resuspended at a concentration of 2×10^6^ cells/mL with RPMI 1640 containing 5% inactivated FBS. TC-1 cells used as target cells were suspended at 2×10^5^ cells/mL. Spleen and target cells were added to 96-well plates at an effector cell/target cell (E∶T) ratio of 2.5∶1, 5∶1, 10∶1, or 20∶1 and then incubated for 4 h at 37°C. The supernatant (50 µL) was transferred to enzymatic assay plates and then added to 50 µL of reconstituted substrate mix in each well. After 30 min of incubation at room temperature, 50 µL of stop solution was added to each well. The release of LDH in the supernatants from killed cells was measured at 490 nm absorbance according to the manufacturer's protocol. The percent cytotoxicity was calculated as % Cytotoxicity = (Experimental−Effector Spontaneous−Target Spontaneous)/(Target Maximum−Target Spontaneous)×100.

### Antibody assay

Specific anti-E7E6 or CTLA-4 serum IgG in mice was determined using ELISA. Briefly, microtiter plates (Corning Inc, USA) were coated overnight at 4°C with 0.5 µg/well recombinant E7E6 protein (produced by Sangon, Shanghai, China) or recombinant CTLA-4 protein (produced by Sino Biological Inc, China) in 100 µL of carbonate buffer (pH 9.6) and then blocked with PBST (PBS with 0.05% Tween 20, Sangon, China) containing 3% bovine serum albumin for 2 h at 37°C. Serially diluted mouse sera in PBST were added to the plates for 1 h at 37°C and then incubated with 1∶10000 dilution of goat anti-mouse IgG (Thermo, USA) for 1 h at 37°C. Bound antibodies were determined by 1∶10000 diluted HRP-conjugated donkey anti-goat IgG (Thermo, USA) for 1 h at 37°C. The antigen-antibody complex was detected with OPD-phosphoric-citric acid buffer substrate at OD 490 nm.

### Statistical analysis

SPSS20.0 for Mac (SPSS, USA) was used for statistical analysis. The survival rates of the mice were determined by Kaplan–Meier analysis. Differences in anti-E7E6 specific antibodies and E7E6 specific cytotoxicity among the groups were determined by one-way ANOVA.

## Results

### Expression of E7E6 protein in 293 cells

Wild-type E6, recombinant CTLA4-E7E6, or E7E6 fusion protein was detected in the 293 cells transfected by pCTLA4-E7E6 or pE7E6 with mouse anti-HPV16 E7 antibody (Figure 2). The fusion proteins did not downregulate the expression of p53 or pRb in the 293 cells compared with wild-type E7 or E6 expression plasmids (Figure 2). This result indicates that the oncogenicity of E7 and E6 in recombinant fusion proteins was disrupted in 293 cells. Interestingly, wild type E7E6 or CTLA-4 and wild-type E7E6 fusion protein also did not downregulate the expression of p53 and pRb. The C-terminal of E7 directly fused with the N-terminal of E6. Thus, the functions of E7 and E6 may be disrupted.

### Binding of CTLA4-E7E6 fusion protein to DCs

The results of ELISA assay showed that the concentration of recombinant CTLA4-E7E6 fusion protein in the culture medium of the 293 cells transfected with pCTLA4-E7E6 was 0.16 ng/µL. CTLA4 is a B7 molecule ligand that is expressed on the surface of APCs, including DCs. FACS analysis showed that the culture medium containing recombinant CTLA4-E7E6 fusion protein induced a remarkable shift in mean fluorescence intensity compared with the controls of pE7E6 or pVAX1-transfected cells (42.91 vs. 7.55 and 2.86, Figure S1). This result indicates that CTLA4-E7E6 fusion protein can bind to DCs and facilitate antigen capture.

### Therapeutic immunization retarded tumor development in mice

**Figure 4:**
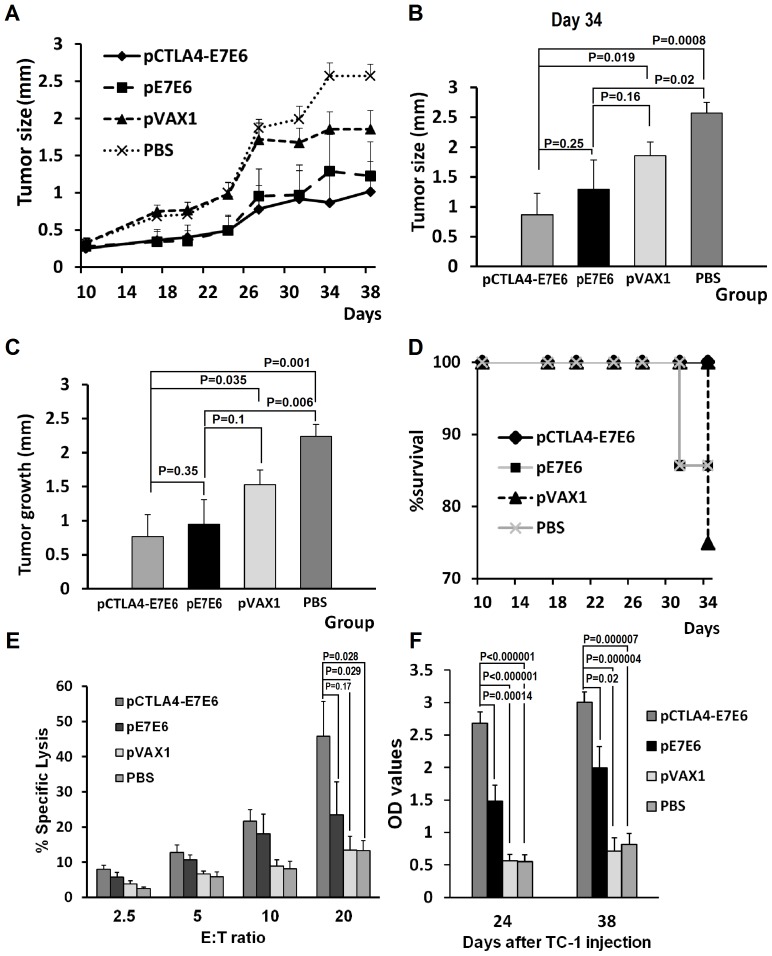
Anti-tumor therapeutic efficacy of pCTLA4-E7E6. (**A**) Effects on tumor growth by therapeutic vaccination. Tumor-carrier mice were vaccinated with pCTLA4-E7E6, pE7E6, pVAX1 or PBS twice on day 10 and 17 after TC-1 cells injection. Tumor size was recorded twice or thrice a week. (**B**) Statistic analysis of tumor sizes on day 34 among groups. The graph shows means ± SE. (**C**) Statistic analysis of tumor development between day 10 and day 38 among groups. (**D**) Kaplan-Meier survival analysis of mice survival in different groups. (**E**) Tumor specific CTL responses. Spleen cells were collected from alive mice in different groups on day 38 and used as effector (E) cells. TC-1 cells were used as target (T) cells. The cellular immune response against E7E6 was determined by lactate dehydrogenase (LDH) release assay. Various E/T ratios were tested as indicated. The data were presented as means ± SE. (**F**) The levels of specific anti-E7E6 serum IgG were determined by ELISA assay on day 24 and 38. The antibody levels of each group were presented as mean OD values ± SE.

Mice with TC-1 tumor cells were developed to assess the anti-tumor therapeutic efficacy of the DNA vaccines pCTLA4-E7E6 and pE7E6. Vaccination was conducted when tumors were established on day 10 and boosted on day 17. Group pCTLA4-E7E6 showed significantly smaller tumors than control groups (Group pVAX1 and Group PBS) at almost all examination time points after day 10 (*P*<0.05), as presented in Figure 4B (day 34). However, Group pE7E6 only showed significantly smaller tumors than Group PBS at all time points (*P*<0.05) (Figure 4A). The tumor growth between days 10 and 38 was calculated to analyze the growth rate of tumors. Group pCTLA4-E7E6 showed significantly slower tumor growth than the two control groups (*P* = 0.035 or 0.001, respectively). However, Group pE7E6 only showed significantly slower tumor growth than Group PBS (*P* = 0.006; Figure 4C).

In terms of protection outcomes, the survival rates of Group pCTLA4-E7E6, Group pE7E6, Group pVAX1, and Group PBS after the experiment were 100%, 85.7%, 75%, and 85.7%, respectively (Figure 4D). Although Kaplan–Meier analysis showed no significant difference among the groups, only the mice in Group pCTLA4-E7E6 were completely survived the tumor challenge (Figure 4D). Overall, pCTLA4-E7E6 showed relatively stronger anti-tumor effects than pE7E6 in therapeutic immunization.

We analyzed cellular and humoral immune responses against HPV16 to further evaluate the protection responses induced by DNA vaccination. Group pCTLA4-E7E6 showed significantly higher CTL-mediated lysis against the TC-1 cells than the two control groups (*P*<0.05, E∶T = 20∶1). Group pCTLA4-E7E6 showed higher CTL responses than Group pE7E6 without statistical significance (*P* = 0.172, E∶T = 20∶1) (Figure 4E). Group pCTLA4-E7E6 showed significantly higher anti-E7E6 specific antibodies than all other groups on days 24 and 38 (*P*<0.05). Overall, pCTLA4-E7E6 induced stronger immune responses than pE7E6 in therapeutic immunization.

### Preventive immunization protected mice from tumor development

**Figure 5:**
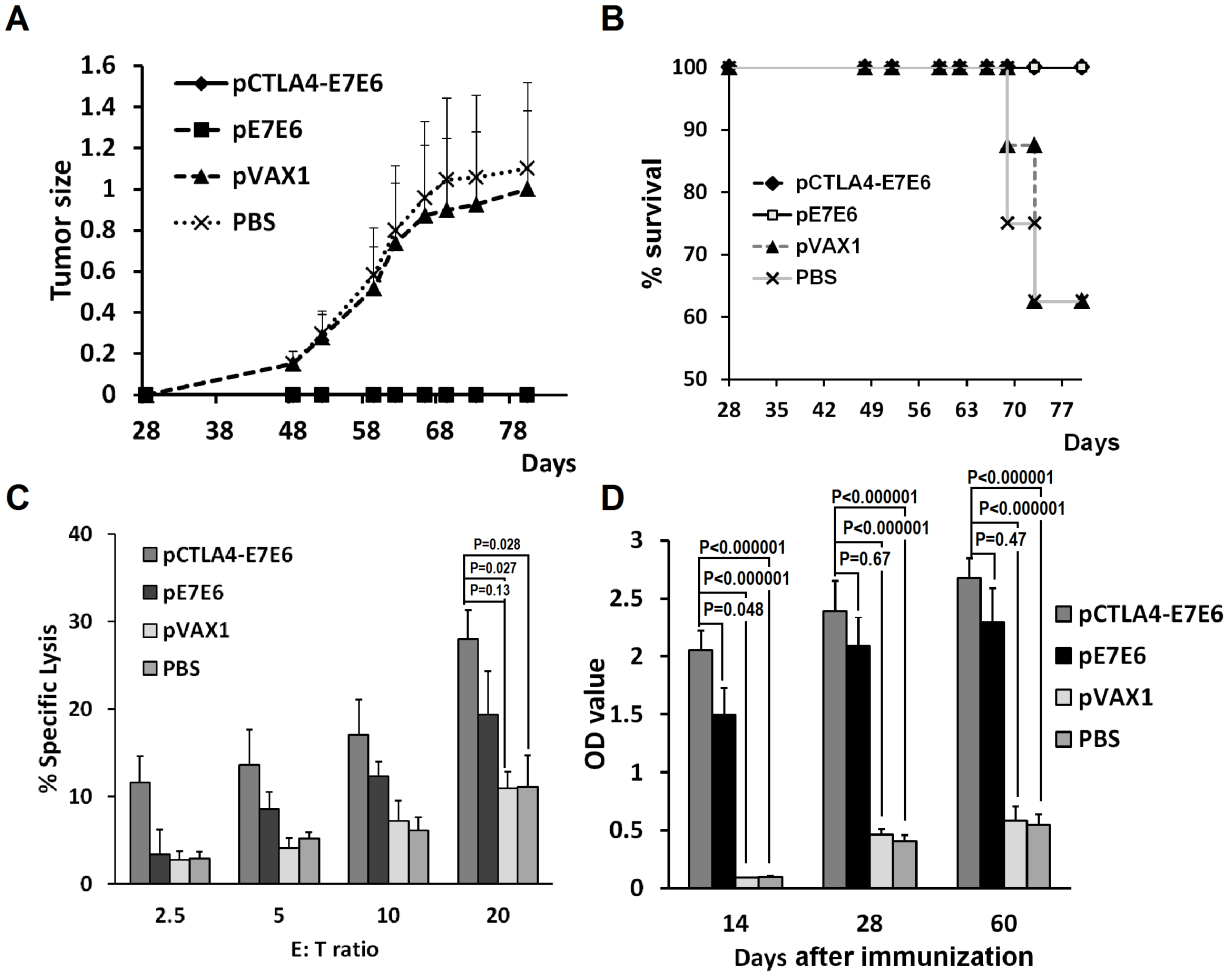
Anti-tumor preventive efficacy of pCTLA4-E7E6. (**A**) Effects on tumor growth by preventive vaccination. Mice were vaccinated with pCTLA4-E7E6, pE7E6, pVAX1 or PBS twice on day 0 and 14. On day 28 mice were challenged with 6×10^4^ TC-1 tumor cells. Tumor size was recorded twice or thrice a week. (**B**) Kaplan-Meier survival analysis of mice survival in different groups. (**C**) Tumor specific CTL responses. Spleen cells were collected from alive mice in different groups on day 80 and used as effector (E) cells. TC-1 cells were used as target (T) cells. The cellular immune response against E7E6 was determined by lactate dehydrogenase (LDH) release assay. Various E/T ratios were tested as indicated. The data were presented as means ± SE. (**D**) The levels of specific anti-E7E6 serum IgG were determined by ELISA assay on day 14, 28, and 60. The antibody levels of each group were presented as mean OD values ± SE.

After therapeutic immunization, the preventive efficiency of pCTLA4-E7E6 or pE7E6 was also determined. After twice immunization, the mice were challenged with TC-1 tumor cells. The two DNA vaccination groups received complete protection from tumor cell challenge during the entire experimental period. By contrast, the two control groups developed tumors (Figure 5A). Approximately 62.5% of the mice from control groups survived tumor cell challenge on day 80. The results of Kaplan–Meier analysis showed that the two control groups had significantly lower survival rate than the two DNA vaccination groups (*P*<0.05). These results indicate that pre-immunization with E7E6 DNA vaccine can completely protect mice from low-dosage tumor cell challenge.

No difference in protection efficiency was observed between Group pCTLA4-E7E6 and Group pE7E6; however, Group pCTLA4-E7E6 generated significantly more anti-E7E6 specific antibodies than Group pE7E6 on day 14 (Figure 5D). The two DNA vaccination groups generated significantly more anti-E7E6 specific antibodies than the control groups (*P*<0.00001). Group pCTLA4-E7E6 also showed stronger CTL responses than Group pE7E6 without statistical significance (*P* = 0.132, E∶T = 20∶1) (Figure 5C). The two DNA vaccination groups showed significantly stronger CTL responses than the control groups (*P*<0.05). Overall, pCTLA4-E7E6 induced stronger immune responses than pE7E6 in preventive immunization.

## Discussion

HPV-preventive vaccines are highly effective in blocking viral entry but are generally ineffective in eliminating existing infections. Thus, developing therapeutic vaccines to treat patients with established HPV infection or HPV-associated malignancies is still urgently needed. During the past decade, many therapeutic vaccines have been tested in clinical and preclinical experiments, including proteins, peptides, DNA vaccines, and recombinant viruses. For example, Einstein *et al.* [21] used a fusion protein, HspE7, which contains heat shock protein (Hsp65) and HPV16 E7 in a clinical trial. Approximately 22.5% or 55% of patients with CIN III immunized with HspE7 showed complete or partial pathologic regression response, respectively. Another study using the same vaccine achieved complete pathologic regression in 35.5% of patients with high-grade CIN [22]. In a multicenter phase II trial, 17% of patients showed clinical responses after vaccination with a recombinant fusion protein comprising HPV 16, E6/E7/L2 (TA-CIN) as a primer vaccine, and a recombinant vaccinia virus encoding HPV 16 and 18 E6/E7 (TA-HPV) as a boost vaccine [23]. A clinical trial using a DNA vaccine (ZYC-101a) encoding CTL epitopes against E6 and E7 of HPV 16 and HPV 18 showed that the resolved proportion in the ZYC101a group is higher than that in the placebo group(43% versus 27%), but these results show no statistical significance [24]. Another phase I clinical trial with HPV16 DNA vaccine expressing a Sig-E7(detox)-Hsp70 fusion protein showed completely histological regression in three out of nine patients in the highest-dose cohort, although the difference is not significant compared with the unvaccinated cohort [25]. These clinical studies have encouraged other studies. However, efficient therapeutic vaccines still need to be conducted to control HPV-related malignant diseases.

Strengthening immune responses against HPV E6 and E7 is the key to control the progress of HPV-associated malignant diseases. In the present study, CTLA-4 fusion DNA vaccine was used to enhance specific anti-HPV16 E7E6 immune responses. CTLA-4 or CD152 is principally expressed on the surface of helper T-cells (Th). CTLA-4 is the receptor of B7 molecules mainly expressed on the surface of APCs. Boyle *et al.* [26] fused CTLA-4 with human IgG and dramatically strengthened immune responses against human IgG. CTLA-4 was also fused with antigens of *Streptococcus mutans*, a well-documented cariogenic bacterium, and significantly enhanced specific immune responses and anti-caries effects [12]. In the present study, CTLA-4 was used to develop anti-HPV DNA vaccines. Similar to our previous study, the present study showed that CTLA-4 significantly enhanced humoral immune responses against HPV16 E7E6 in both preventive and therapeutic immunization experiments. Furthermore, pCTLA4-E7E6 showed relatively stronger specific CTL responses and anti-tumor effects than pE7E6 based on the results of therapeutic immunization experiment.

CTLA-4 is a negative regulator of immune responses. In the present study, the signal peptide and extracellular domains of human CTLA-4 were fused with E7E6. The CTLA-4 fragment bound to CD80 and CD86, and negatively regulated immune responses. Human CTLA-4 also induced anti-CTLA-4 antibody (Figure S2) and may promote anti-tumor immune responses. Many studies have reported that the negative regulatory function of CTLA-4 might be blocked by anti-CTLA-4 antibody. Tumor growth may be suppressed by either DNA vaccines against CTLA-4 or purified specific anti-CTLA-4 monoclonal antibody [27], [28]. Thus, the mechanism by which CTLA-4 fusion DNA vaccine enhances immune responses needs further exploration.

Zheng *et al.* [29] attempted to utilize CTLA-4 to enhance anti-HPV immune responses. They found that a recombinant fusion protein consisting of extracellular regions of CTLA-4 and HPV16 E7 can generate more specific antibodies and stronger specific CTL responses than the E7 protein alone. In the present study, a DNA vaccine encoding a fusion protein was constructed by fusing the extracellular region of CTLA-4 with both E6 and E7 genes. DNA vaccines generally display stronger and longer cellular anti-tumor immune responses than protein vaccines.

In tumor therapy treatment, cytotoxicity response is crucial in killing tumor cells. Aside from T-cell mediated cytotoxicity, antibody-dependent cell-mediated cytotoxicity (ADCC) has also attracted attention in the field of tumor therapy [30]. Therapeutic antibodies against tumors specifically bind and lysate tumor cells through ADCC or complement-mediated cytolysis [31]. Neutrophils [32], macrophages, and natural killer cells [33] reportedly express Fc receptors and mediate antibody-induced anti-tumor effects. Anti-E7E6 antibody may identify E7 or E6 peptides presented on the surfaces of tumor cells and initiate ADCC to kill tumor cells. Increased specific anti-E7E6 antibody induced by pCTLA4-E7E6 may enhance its anti-tumor effects.

In summary, fusing CTLA-4 with E7 and E6 can enhance cellular and humoral immune responses against HPV16. This strategy may provide useful ideas for developing therapeutic HPV DNA vaccines. However, further studies are needed to optimize the strategy.

## Supporting Information

Figure S1 **FACS analysis of the binding ability of CTLA-4-E7E6 fusion protein to mouse dendritic cell DC2.4.** Cells were incubated with the supernatants of cultured media from the 293 cells transfected with pCTLA4-E7E6, pE7E6, or pVAX1. The binding of the CTLA4-E7E6 fusion protein to cells was detected by FITC labeled goat anti-human IgG antibody (Sigma) and analyzed by FACS. Geometric mean fluorescence intensities of cells transfected by pCTLA4-E7E6, pE7E6, and pVAX1 were 42.91, 7.55, and 2.86 respectively.
(TIF)

Figure S2 **Anti-CTLA-4 antibody levels in therapeutic (A) or preventive (B) vaccination.** The levels of specific anti-CTLA-4 serum IgG were determined by ELISA assay. The antibody levels of each group were presented as mean OD values ± SE.
(TIF)
