# Supplementary figures and images for: Fusion of CTLA-4 with HPV16 E7 and E6 Enhanced the Potency of Therapeutic HPV DNA Vaccine
Source: PLoS One. 2014 Sep 29;9(9):e108892. doi: 10.1371/journal.pone.0108892 (PMC4181872; doi:10.1371/journal.pone.0108892)

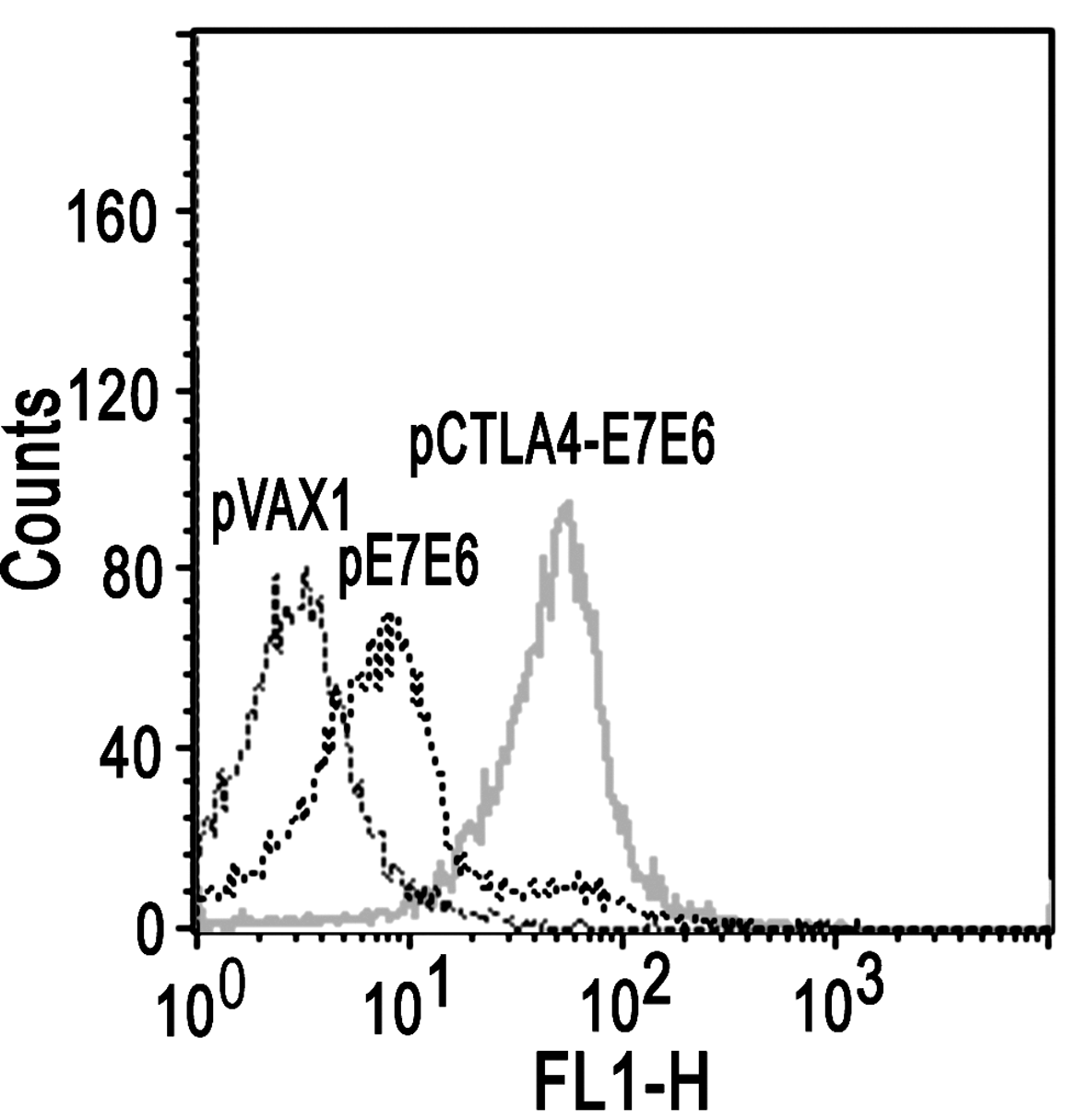

Supplement: Figure S1 — FACS analysis of the binding ability of CTLA-4-E7E6 fusion protein to mouse dendritic cell DC2.4. Cells were incubated with the supernatants of cultured media from the 293 cells transfected with pCTLA4-E7E6, pE7E6, or pVAX1. The binding of the CTLA4-E7E6 fusion protein to cells was detected by FITC labeled goat anti-human IgG antibody (Sigma) and analyzed by FACS. Geometric mean fluorescence intensities of cells transfected by pCTLA4-E7E6, pE7E6, and pVAX1 were 42.91, 7.55, and 2.86 respectively. (TIF) [file pone.0108892.s001.tif]

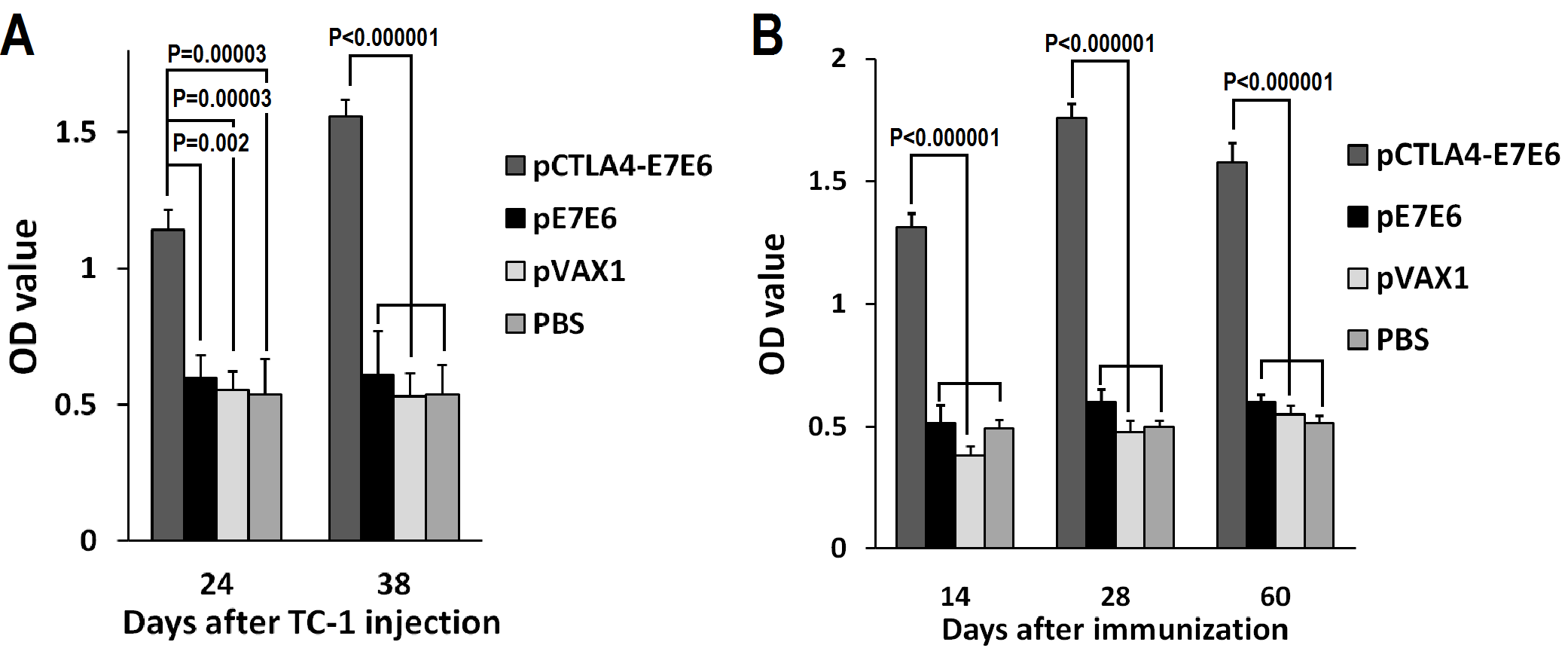

Supplement: Figure S2 — Anti-CTLA-4 antibody levels in therapeutic (A) or preventive (B) vaccination. The levels of specific anti-CTLA-4 serum IgG were determined by ELISA assay. The antibody levels of each group were presented as mean OD values ± SE. (TIF) [file pone.0108892.s002.tif]
